# Effect of pH-Regulation on the Capture of Lipopolysaccharides from *E. coli* EH100 by Four-Antennary Oligoglycines in Aqueous Medium

**DOI:** 10.3390/ma14247659

**Published:** 2021-12-12

**Authors:** Anna Y. Gyurova, Kaloyan Berberov, Alexander Chinarev, Ljubomir Nikolov, Daniela Karashanova, Elena Mileva

**Affiliations:** 1 Institute of Physical Chemistry, Bulgarian Academy of Sciences, 1113 Sofia, Bulgaria; any_gyurova@abv.bg (A.Y.G.); kaloyan.ber@abv.bg (K.B.); ljubo@ipc.bas.bg (L.N.); 2 Shemyakin-Ovchinnikov Institute of Bioorganic Chemistry, Russian Academy of Sciences, 117997 Moscow, Russia; chinarev@carb.ibch.ru; 3 Institute of Optical Materials and Technologies, Bulgarian Academy of Sciences, 1113 Sofia, Bulgaria; dkarashanova@yahoo.com

**Keywords:** lipopolysaccharides, antennary oligoglycines, tectomers, surface tension, surface dilational rheology, microscopic foam films

## Abstract

Bacterial lipopolysaccharides (LPS) are designated as endotoxins, because they cause fever and a wide range of pathologies in humans. It is important to develop effective methodologies to detect trace quantities of LPS in aqueous systems. The present study develops a fine-tuning procedure for the entrapment of trace quantities of LPS from *E. coli* EH100. The capture agents are self-assemblies (tectomers) formed by synthetic four-antennary oligoglycine (C-(CH_2_-NH-Gly_7_)_4_, T4). Based on previously performed investigations of bulk and adsorption-layer properties of aqueous solutions containing T4 and LPS, the optimal conditions for the entrapment interactions are further fine-tuned by the pH regulation of aqueous systems. A combined investigation protocol is developed, including dynamic light scattering, profile analysis tensiometry, microscopic thin-liquid-film techniques, and transmission electron microscopy. The key results are: (1) two types of complexes between T4 and LPS are generated—amphiphilic species and “sandwich-like” hydrophilic entities; the complexes are smaller at lower pH, and larger at higher pH; (2) an optimum range of pH values is established within which the whole quantity of the LPS is entrapped by the tectomers, namely pH = 5.04–6.30. The obtained data substantiate the notion that T4 may be used for an effective capture and the removal of traces of endotoxins in aqueous systems.

## 1. Introduction

The detection and capture of lipopolysaccharides (LPS) in aqueous media are important challenges, related to human health issues. LPS are the key components of the outer surface layer of the cell walls in Gram-negative bacteria, like the human pathogens *Escherichia coli (E. coli)* [1,2,3,4,5,6]. LPS contribute to the outer membrane integrity and provide a barrier, which effectively protects the bacteria from hostile environments (ions, antimicrobial compounds, antibiotics, etc.) [5,7,8,9,10,11]. In addition, LPS are known to be highly immunogenic, because they carry antigenic determinants capable to activate the innate immunity. This alerts the host about the invasion of Gram-negative bacteria at very early stages; however, sometimes the results are uncontrolled pathological responses such as fever, hypotension, and septic shock (the tenth leading cause of death in high-income countries) [12]. LPS remain toxic even when they are released upon bacteria disintegration, thus inducing an acute inflammatory response at higher concentrations, or the development of certain chronic diseases at lower concentrations [4,13,14,15,16]. The lethal concentration of LPS for humans in aqueous media is very low [5,6,17,18,19,20], and that is why they are referred to as bacterial endotoxins [1,7].

**Scheme 1:**
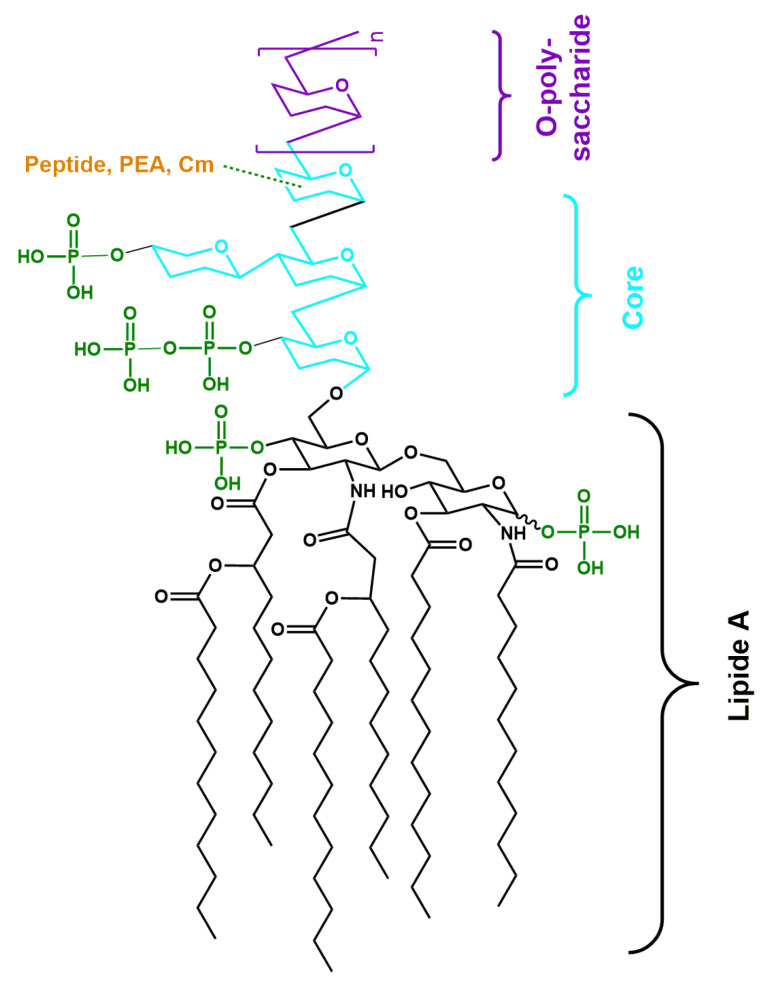
Schematic presentation for the general structure of lipopolysaccharide (LPS).

LPS have a tripartite structure (Scheme 1). The hydrophobic portion (Lipid A) consists of a bisphosphorylated β-1,6 glucosamine disaccharide backbone substituted via ester and amide bonds with a variable number (3–7) of fatty acids. Hydrophilic fragments of LPS are presented by a core moiety (inner and outer core) attached to lipid A (a branched oligosaccharide structure built up from 10–15 sugar units, and heavily decorated with mono- and diphosphate, phosphatidylethanolamine (PEA), carbamoyl (Cm) residues, peptide groups), and with an O-antigen chain polysaccharide containing various numbers of repeating units, in turn made up of 1–10 sugar residues (Scheme 1). Due to phosphorylation and the presence of specific sugars (viz. heptulosonic and nonulosonic acids) in the inner core, LPS molecules are negatively charged [1,5,7,19]. In the absence of outer O-specific component, Ra to Rd mutants may be distinguished. While Rd LPS (“deep rough” mutants) contain only inner polysaccharide cores, the Ra LPS (“rough” mutants) have longer polysaccharide segments [1,2,18,19].

The presence of both hydrophilic and hydrophobic portions determines the amphiphilic nature of the LPS molecules [1,2]. Thus, LPS can self-aggregate in aqueous solutions into supramolecular structures with different shapes and sizes [4,19]. The formation of micelles (above the critical micelle concentration CMC), or LPS layers at hydrophobic surfaces, incl. air/solution interfaces (at concentrations lower than CMC), and the onset of premicelles (at the so-called apparent critical micelle concentration CMC_a_), have been reported in Refs. [4,21,22]. Exposed at the interface between the bacterium and its environment, LPS is responsible for the *E.coli* negative electric charge in aqueous solutions. Therefore, changes in the pH of the aqueous media could affect the electrostatic interactions between LPS molecules and charged particles and/or interfaces.

There are various approaches for LPS detection in fluid media (see e.g., [23,24,25]). One should mention the rabbit pyrogen test, which involves injecting the biological sample in question into live rabbits, and waiting for a fever to develop [23]. Among the methods currently widely used is the *Limulus polyphemus* amoebocyte lysate assay (LAL); it is based on a coagulation cascade triggered by endotoxins, and involves three serine proclotting enzymes [23,24]. An animal-free variant of the assay has been introduced in practice; it uses the recombinant Limulus proenzyme Factor C and its fluorogenic synthetic peptide substrate [24,26,27]. In attempts to modernize endotoxin detection methods, biosensor techniques (based on electrochemical, optical, magnetic, surface plasmon resonance and other approaches) are developing; to compose sensor platforms, peptide, proteins (enzymes and antibodies), aptamers and cells are used as sensing elements [24,25].

Due to the adverse effects of endotoxins on human health, particular attention has been paid to the advancement of effective procedures for the detection of LPS in aqueous systems. Thus, the traditional large scale purification approaches, such as coagulation, sedimentation, filtration (soil, sand and etc.), activated carbon absorption, biological treatment, chemical disinfection (chlorination, oxidation and ozonation) and treatment with UV, used alone or in combination with each other, give rise to noticeable reductions in levels of endotoxin contaminations in drinking and reclaimed water [26]. Biopharmaceutical purification is another area that is in critical need of technologies to remove even trace contamination with endotoxins of water. The list of the most used methods encompasses, e.g., ultrafiltration, affinity adsorption chromatography, two-phase extraction, plasma discharge [26,27,28]. However, to the best of our knowledge, there is not a general capture procedure that might commonly fit most of the purification scenarios. So, further research is desirable for finding cost-effective and finely tunable approaches that could reduce the biopharmaceuticals losses, while eliminating traces of LPS contaminants in aqueous systems.

Recently, a novel approach has been proposed for the identification of ultralow quantities of LPS [29,30]. It was established that LPS from Ra-strain of *E. Coli* EH100 in aqueous solutions can be captured by positively charged hydrophilic nanoplatforms (tectomers), formed by four-antennary oligoglycine molecules C-(CH_2_-NH-Gly_7_)_4_ (T4). It was shown that, at low endotoxin concentrations, the whole quantity of LPS can be caught by the tectomers. The entrapment is due to electrostatic interactions between the positively charged oligoglycine self-assemblies and the negatively charged LPS entities, resulting in the onset of surface-active complexes. These complexes are detected through surface tension measurements and by microscopic foam films. The proposed system formulation, encompassing oligoglycine tectomers and LPS traces, has a high potential for the additional fine-tuning of the capture phenomena, e.g., through changes in pH of the aqueous medium, temperature variations, addition of low-molecular mass (LMM) electrolytes, etc.

The symmetric four-antennary oligoglycine (C-(CH_2_-NH-Gly_7_)_4_, T4) is a synthetic compound [31,32,33] which has recently been investigated in more details [29,30,34]. The structure of these highly symmetric molecules offers specific options for the spontaneous onset of intra- and intermolecular hydrogen-bonding network at a high cooperativity level, known as Polyglycine II (PGII) [35,36,37,38]. The supramolecular PGII packing patterns predetermine the formation of flat (discotic) bulk nanoaggregates (tectomers) in the aqueous T4 solutions [31,32,34]. Because of the PGII motifs, which result in ‘click-clack’ (steric) interactions, the self-assembled structures are also stable at ambient temperature. The bulk self-assembly phenomenon was systematically investigated in the course of 24 h, and within a concentration range of more than two orders of magnitude, using the DLS technique [34]. The presence of terminal amine groups in the T4 antennae presumes that the four-antennary oligoglycine aqueous systems can have a considerable pH-sensitivity, which will inevitably influence both the T4 aggregation phenomena, and will affect the size distribution of the obtained tectomers in the aqueous medium.

The goal of the current study is to perform a systematic investigation on the pH-regulation of the self-assembling tendencies of T4 and of LPS in aqueous solutions at ambient temperature. The aim is to collect sufficient data so as to clarify the impact of changes in pH values on the possible entrapment of trace quantities of lipopolysaccharides from *E. coli* EH100 by the oligoglycine tectomeric aggregates. The final objective is to elucidate how the possible modification of the four-antennary oligoglycine self-assembling tendencies due to the specific pH of the aqueous systems permits the additional fine-tuning of the LPS capture phenomena.

## 2. Materials and Methods

The investigated systems are aqueous solutions of lipopolysaccharides from *Escherichia coli* EH100 (LPS) of four-antennary oligoglycine (T4), and of their mixtures (T4+LPS). Doubly distilled water is used for their preparation. T4 is synthesized in the Laboratory of Carbohydrates in the Institute of Bioorganic Chemistry, Russian Academy of Sciences, Moscow, Russia and is supplied in a salt form ([Gly_7_-NHCH_2_]_4_C*4HCl). LPS are supplied by Merck KGaA, Darmstadt, Germany (Ra mutant, purity ≥97%, purified by phenol extraction). The LPS aqueous solutions are prepared with an initial concentration of 5 µg/L, and stored at 4 °C for two months. The investigated LPS aqueous samples are obtained from the stock solution, and the final LPS concentration in all measurements is 0.5 µg/L. LPS sample is added to the T4 solutions initially kept for 24 h, and the experiments are performed without further incubation.

Test experiments show that the self-assembly in aqueous solutions within the T4 concentration range investigated in [29,30,34] has no significant dependence on the ‘native’ pH (in doubly distilled water, without pH-regulation) when varying the oligoglycine quantity. In these cases, the pH values change from pH 6.0 (5 × 10^−5^ mol/L) to pH 5.5 (1 × 10^−3^ mol/L). This suggests that both the monomers and the tectomers are positively charged due to the existence of protonated terminal amino groups (NH_3_^+^). Indeed, the recorded electrophoretic mobility is ~2.5 µm.cm/V.s, and it is not significantly time-dependent within the incubation period of 24 h (as shown in Figure 10 of [34]). In the present investigation, the oligoglycine concentration is fixed at 2 × 10^−4^ mol/L. The concentration corresponds to the optimal T4 quantity for capturing the whole LPS quantity at the ‘native’ pH (pH = 5.70), as established in [30].

Britton–Robinson universal buffer is applied to maintain the pH of the samples [39]. The buffer consists of solutions of boric acid (H_3_BO_3_, Merck KGaA, Darmstadt, Germany, purity ≥99.97% (trace metal basis)), phosphoric acid (H_3_PO_4_, Merck KGaA, Darmstadt, Germany, purity ≥99.999% (trace metal basis)) and acetic acid (CH_3_COOH, Merck KGaA, Darmstadt, Germany, purity ≥99.99% (trace metal basis)), in a ratio 1:1:1 by volume. The components are mixed with different amounts of sodium hydroxide (Merck KGaA, Darmstadt, Germany, NaOH, purity ≥97% (titration by HCl)) to obtain the various pH values. The investigated pH range is 3.8–10.0. The temperature is kept strictly at 20.0 ± 0.1 °C, with the samples either retained in the thermostatic chamber of the measuring instrumentation, or in another thermostatic device between the separate measurements.

The examination of the bulk solution properties is performed by dynamic light scattering instrumentation (DLS, Zetasizer Nano ZS, Malvern Instruments Ltd. Malvern, UK). The scattering angle is 173° (non-invasive backwards scattering). The aggregate size distributions by volume and by number are recalculated using Mie theory [40,41]. The electrophoretic mobility is recorded by the method of electrophoresis.

Some test experiments are also performed by transmission electron microscopy (TEM), using the technique of HR STEM (JEOL JEM 2100, JEOL Ltd., Tokyo, Japan). The preparation procedure is the following: one drop of each solution is fixed on a carbon coated standard Cu TEM grid. The grids are left to dry in a dust-free environment at room temperature, and then introduced into the microscope chamber for the morphology analysis of the samples.

The adsorption layer properties at the air/solution interface are investigated by the emerging-bubble option of the profile analysis tensiometry (PAT-1, Sinterface, Berlin, Germany) [42,43,44]. The measurements are performed in the course of 48 h. Surface dilational rheology is determined through oscillations within the low-frequency range of 0.005–0.2 Hz; the oscillation amplitudes are in the range of 5–10% of the bubble surface area.

The formation and drainage behaviors of microscopic foam films are studied by the microinterferometric thin-liquid-film techniques (TLF) equipped with the Scheludko-Exerowa cell [45,46]. The film experiments begin with an initial 2 h thermal equilibration at 20.0 ± 0.1 °C.

## 3. Results

### 3.1. Effect of pH on the Bulk Self-Assembly Phenomena

**Figure 1:**
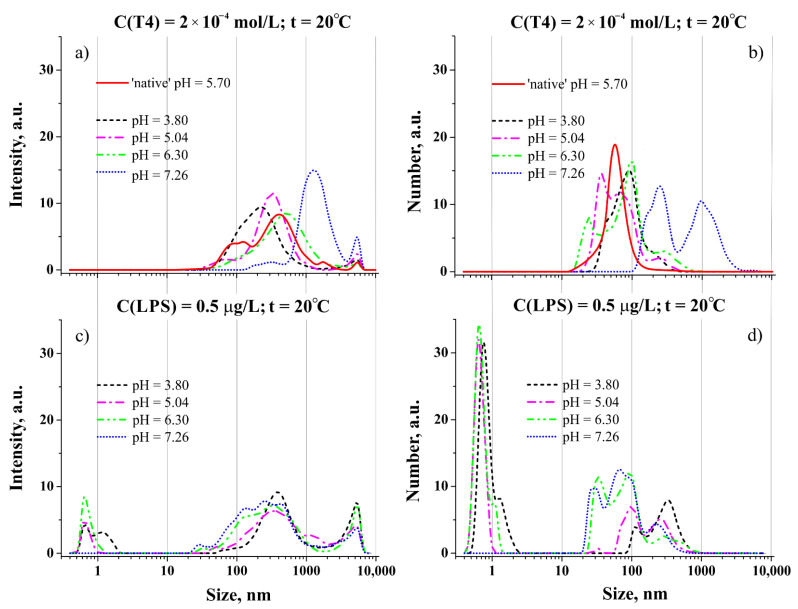
Mean size distributions in aqueous solutions with different pH, by intensity and by number. (**a**,**b**) for T4 at C(T4) = 2 × 10^−4^ mol/L; (**c**,**d**) for Ra LPS from *E. coli* EH100 at C(LPS) = 0.5 µg/L. The incubation time of T4 solutions is 24 h. LPS solutions are prepared from a stock solution, stored at 4 °C for 2 months. The temperature is kept constant at t = 20 °C.

**Figure 2:**
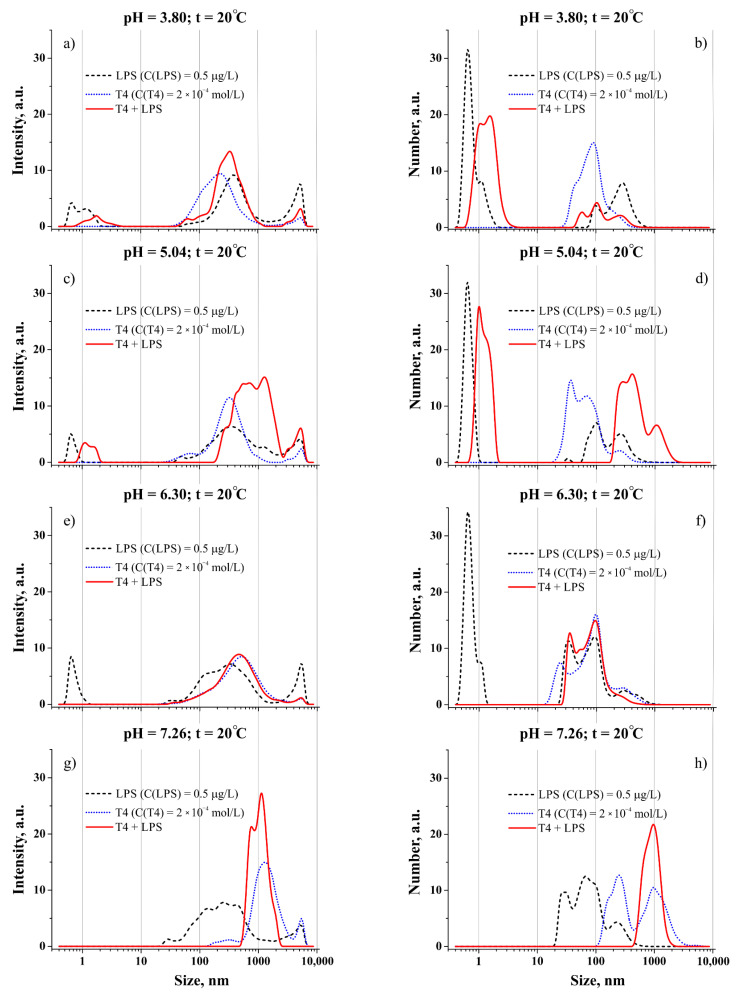
Mean size distributions by intensity of the scattered light and by number for buffered aqueous solutions of T4 at concentration C(T4) = 2 × 10^−4^ mol/L, of Ra LPS from *E. coli* EH100 at concentration C(LPS) = 0.5 µg/L, and of mixed solutions of T4 and LPS. The incubation time of T4 solutions is 24 h; incubation time of LPS solutions is 2 months. Temperature is t = 20 °C. (**a**,**b**) pH = 3.80; (**c**,**d**) pH = 5.04; (**e**,**f**) pH = 6.30; (**g**,**h**) pH = 7.26.

The results from the DLS study on the effect of the pH values for each of the investigated aqueous solutions are presented in Figure 1a–d and Figure 2a–h. Note that only data about pH = 3.80–7.26 are displayed, because at higher pH values, the systems become opaque, and upon further increase of the pH, a massive sedimentation is observed. In the latter cases, the pH of the supernatant remains at ~8.0, even for pH = 10.0.

In Figure 1a,b are shown the DLS data for the averaged size distributions by intensity and by number for T4-only systems, at constant oligoglycine concentration C(T4) = 2 × 10^−4^ mol/L, and for various pH values of the aqueous media. The respective data for the ‘native’ pH = 5.70 are taken from [34] and included for comparison. Due to the prevailing PGII self-assembling motifs, the tectomers in the aqueous solutions of T4 are discotic [31] and quite polydisperse. The method of recalculation for the size distribution by number from the initial results about the size distribution by intensity, refers to Mie theory [40,41]. The recalculation includes a sphericalization step. In case of significant polydispersity, there is a difference in the size distribution by intensity and by number. Here, at lower pH (3.80, 5.04), the difference is less than at higher pH (6.30, 7.26). As is well known, the presentation of the size distribution by intensity is recommended for suspensions rich in larger particles and aggregates, while the presentation by number is more sensitive to entities smaller in size. Insofar as the size distributions of the tectomers are bimodal for all pH values of the buffered solutions, reaching values of ~300 nm and ~1000 nm (for example at pH = 7.26), two types of size distribution are presented. Besides, the bimodal size distribution shifts primarily towards smaller species (~30 nm, ~70 nm for pH = 5.04) and (~20 nm, ~100 nm for pH = 6.30). Unusual mono-modal distribution by number is registered only for the lowest pH (3.80, ~100 nm), and for ‘native’ pH (5.70, ~60 nm). These peculiarities are most probably closely linked to the change (decrease) in the charge density of both the T4 molecules and of the tectomers upon the rise of the solution pH.

The size distributions in the LPS-only systems comprise several modes which also change with the increase in the pH values of the buffer (Figure 1c,d). Overall, the larger-sized LPS nanoaggregates are in the range of ~30–400 nm. As expected, and unlike the T4 solutions, in the LPS case, the size distributions, both by intensity and by number, are shifted towards lower values at higher pH. This is related to the existing negative charges in the LPS molecules. At low pH, these charges are neutralized to a considerable degree as compared to the respective values at higher pH, and it is also possible that premicellar LPS aggregates are present in the solutions. One key observation is the persistent appearance of low-nanometer mode (~1 nm) for all the pH values, except at highest pH = 7.26. These entities may be related to small LPS premicellar species and/or to single LPS molecules. Some larger species (~3000–4000 nm) are detected in the size distributions by intensity, as well. The latter are probably composite clusters, built by several LPS entities during the two-month initial incubation of the stock solution.

In the case of T4+LPS mixtures, the analysis of the size distribution data shows the following (Figure 2a–h):

(i) At low pH (3.80), the bulk T4+LPS nano-species are bi-modal (Figure 2a,b). Note that at pH = 3.80, the respective size distributions are shifted to smaller entities as compared to the respective size distributions of the tectomers in T4-only solutions. This is in synchrony with the respective size distributions in the buffered LPS-only solutions (Figure 1c,d). The data give grounds for the possible hypothesis that the one mode is related predominantly to either LPS-only premicelles, and/or possible smaller T4+LPS composite species containing tiny tectomers. The second mode is most probably due to the formation of larger T4+LPS complexes.

(ii) In the case of pH = 5.04, the size distributions are shifted to larger aggregates as compared to pH = 3.80, because of the tendency for the onset of larger tectomers at higher pH values. This trend might be linked to the enhanced electrostatic interactions of tectomers with both LPS molecules, and the LPS premicellar entities.

(iii) At higher pH (6.30), the bulk size distributions, both by intensity and by number, are definitely moved towards larger species (Figure 2e,f); this phenomenon is in full conformity with the enhanced size distribution of the tectomers in the T4-only solutions. Besides, one might advance the notion that, in fact, all bulk LPS quantities (both single LPS molecules, and LPS premicelles) are entrapped by the T4 nanoplatforms, because the size distributions of the possible T4+LPS complexes coincide with the size distribution in the buffered T4-only solution. As the sizes in the range of ~1 nm are not registered here, one might presume that pH = 6.30 ensures the most suitable conditions for complete entrapment of the LPS quantity by the existing (bulk) tectomers.

(iv) Similar results are obtained for pH = 7.26, but the mean sizes of the aggregates are significantly larger, in full conformity with the size distribution of the tectomers in the T4-only solutions. (Figure 2g,h). Most probably this is due to the formation of T4+LPS complexes, which entrap the LPS entities. It is noteworthy that higher pH values presume the presence of highly charged LPS species, which might not be able to effectively form (pre)micelles. This in turn, might cause single molecules to be more easily captured by the large and positively charged tectomers.

**Figure 3:**
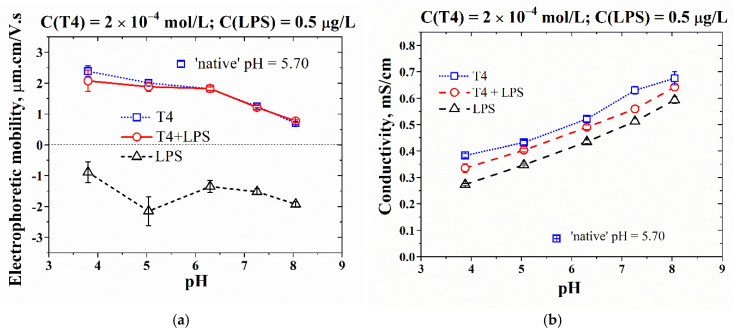
Electrophoretic mobility (**a**) and conductivity (**b**) against the pH values for the buffered aqueous solutions of T4 (C(T4) = 2 × 10^−4^ mol/L), of Ra LPS from *E. coli* EH100 (C(LPS) = 0.5 µg/L), and of mixed T4+LPS solutions. The incubation time of T4 solutions is 24 h; incubation time of LPS solutions is 2 months. The temperature is kept constant at t = 20 °C.

These notions are also supported by the data about the electrophoretic mobility of the species and the conductivity measurements of the investigated samples (Figure 3).

The polydispersity indices PdI of the single-component and mixed solutions are also indicative of being in support of the advanced hypotheses about the specificities in the formation of T4+LPS complexes (Table 1). As a whole, the obtained data provide preliminary hints that the most efficient pH-range in view of complete LPS capture is pH ~5.04–6.30.

**Figure 4:**
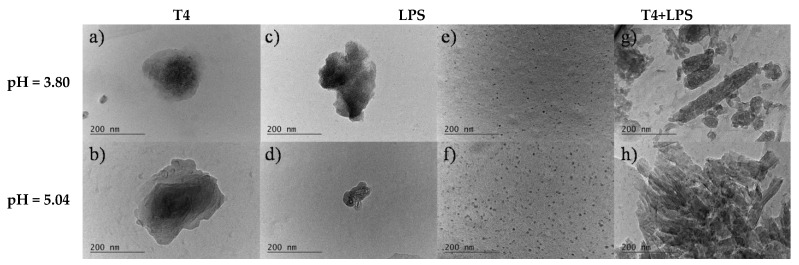
Representative Bright Field (BF) TEM micrographs for buffered aqueous solutions of T4 (C(T4) = 2 × 10^−4^ mol/L) (**a**,**b**), of Ra LPS from *E. coli* EH100 (C(LPS) = 0.5 μg/L) ((**c**,**d**), for the first population), ((**e**,**f**), for the second population), and of mixed system T4+LPS (**g**,**h**) at pH = 3.80 and pH = 5.04. The incubation time of T4 solutions is 24 h; incubation time of LPS solutions is 2 months. The temperature is t = 20 °C.

Further evidence for the advanced hypotheses may be found in the test TEM results, as well. Selected TEM micrographs of the T4 and LPS nanoparticles and T4+LPS complexes formed in the corresponding solutions at different pH values are presented in Figure 4.

It is to be seen that T4 form flat nanoplatforms which are comprised of a few consecutive layers. According to the test TEM results here, the sizes of the individual tectomers vary, as is also demonstrated by DLS measurements (Figure 1a,b), and the diameters measured on the micrographs are predominantly in the interval 20–300 nm. The higher values of the diameters in the DLS results could be related to the formation of composites, as is clearly seen in the TEM results (Figure 4a,b). According to the ТЕМ methodology, the suspensions prepared for analysis are dropped on the solid surfaces of carbon membranes and dried before being placed in a vacuum medium of the microscope. This often leads to a decrease in the particle sizes, and as a result, when measured by TEM images, particles’ diameters tend to have lower values as compared to those obtained by DLS; the particles are in a liquid medium during the DLS measurement procedures. Thus, the T4 species presented in the TEM micrographs have dimensions of ~200 nm for pH = 3.80 (Figure 4a) and ~300–350 nm for pH = 5.04 (Figure 4b). These values coincide well with the particle diameters at the maximum in the size distribution by intensity for both pH values, as presented in Figure 1a.

TEM analysis of the LPS samples at the two different pHs reveals that the morphology of the LPS nanoparticles has two major modes (Figure 4c–f), synchronous with the DLS size distributions, as reported in Figure 1c,d. The LPS particles visualized in Figure 4c,d respectively, have diameters 200–300 nm at pH = 3.80, and 60–100 nm at pH = 5.04. In the first case, the diameter coincides well with the diameter of the particles at the maximum of the size distribution curve by intensity, and in the second case, it is significantly lower (the diameter in the maximum of the size distribution curve by intensity at pH = 5.04 is 200 nm). It should be noted that the particle size distributions at this pH-value are in a broad interval between 100 and 300 nm, and sizes of the order of 100 nm are well-represented in the particles’ ensemble, as seen in Figure 1c. The second population of tiny premicellar nanoparticles, ranging from 1–2 nm in diameter, detected by DLS measurements, is also visualized in Figure 4e,f. For the mixed system T4+PLS at all pH values of the solutions, TEM shows the presence of huge aggregates, which are most probably T4+LPS complexes and composite species of these complexes, with dimensions in the range 3000–4000 nm.

### 3.2. Effect of pH on the Adsorption-Layer Properties at Air/Solution Interface

**Figure 5:**
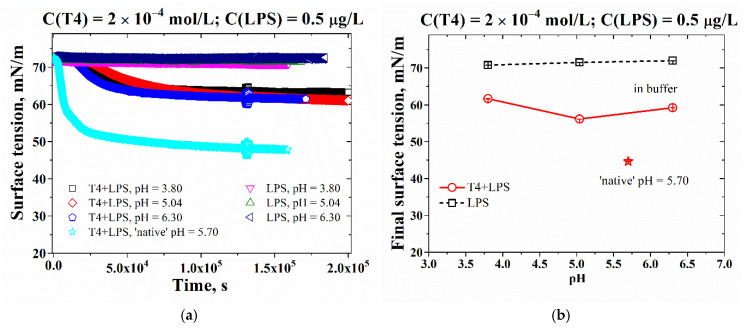
(**a**) Dynamic, and; (**b**) Equilibrium values of the surface tension for of Ra LPS from *E. coli* EH100 at concentration C(LPS) = 0.5 µg/L, and of mixed T4+LPS buffered aqueous solutions with C(T4) = 2 × 10^−4^ mol/L. The initial incubation time of T4 solutions is 24 h; the initial incubation time of LPS stock solution is 2 months. The temperature is t = 20 °C.

The runs of the dynamic surface-tension curves for the aqueous solutions of T4, LPS and their mixtures are presented in Figure 5. The data for the case of ‘native’ pH = 5.70 are also shown for comparison.

In the mixed solutions, and similar to the ‘native’ pH case, the equilibrium surface tension values are achieved within ~13–14 h. The run patterns of the dynamic surface tension curves in the case of mixed T4+LPS solutions, and at the initial moments of formation of adsorption layers at air/solution interface, are reminiscent of the performance usually observed in aqueous solutions of e.g., proteins and of polymer/surfactant mixtures [47,48]. This course of the dynamic surface tension results is usually related to the slow diffusion of high-molecular-mass macromolecules from the bulk of the solution to the interface, as compared to the case of low-molecular-mass surfactants; it might also be due to additional conformational changes upon adsorption at the hydrophobic interface. In the present case, one might consider such behavior as additional evidence for the formation of tectomer/LPS complexes in the bulk of the aqueous solutions, which could adsorb at the air/solution interface. The initial reason for the onset of these complexes is the electrostatic attraction between the positively charged tectomers and the negatively charged LPS species. The obtained results give supplementary grounds that, through the pH regulation of the aqueous systems, the basic changes happen in the bulk of the solution.

Both the single oligoglycine molecules and the tectomers are not surface active in aqueous media, and do not form adsorption layers at the air/solution interface. As shown in Figure 5, the single component LPS system also does not indicate measurable surface activity. However, in all cases of the mixed T4+LPS systems, there is a measurable decrease of the surface tension values. These data are clear signs that surface active complexes are formed in the mixed solutions. This point has already been mentioned in previous studies (Refs. [29,30]), and is further substantiated here also with the well-expressed synchrony of the DLS results, as presented in Figure 2.

The major experimental outcome is that solutions of T4+LPS mixtures do not exhibit essential variations in the recorded dynamic and equilibrium surface tension values within the investigated pH range. Besides, the registered data are considerably higher than in the ‘native’ pH case. Juxtaposed to the results about the structural changes in the bulk of the solutions as presented in Section 3.1, this outcome gives additional evidences that pH-regulation predominantly stimulate the bulk T4 self-assembling tendencies. Overall, the surface tension measurements indicate that the adsorption layer coverage is closely packed and does not change significantly for pH = 3.80–6.30. Open questions however remain, e.g., about the effect of pH changes on the detailed structure of the adsorption layers, and about the reasons why the surface tension values in the cases of buffered solutions are notably higher than for the ‘native’ pH system.

**Figure 6:**
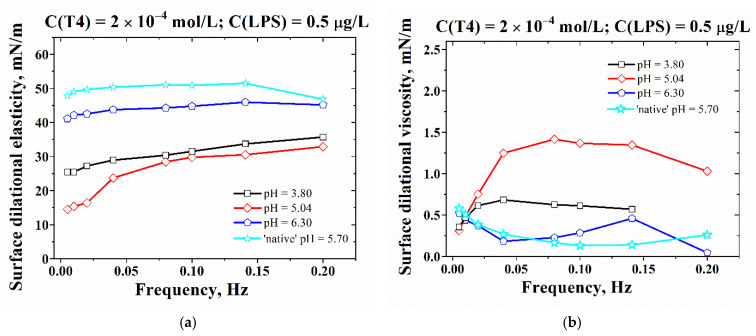
(**a**) Surface dilational elasticities, and; (**b**) Surface dilational viscosities for buffered aqueous solutions of T4+LPS mixtures at various pH values. C(T4) = 2 × 10^−4^ mol/L and C(LPS) = 0.5 µg/L. The initial incubation time of T4 solutions is 24 h; the initial incubation time of LPS stock solutions is 2 months. The temperature is t = 20 °C.

In line with these specific findings are also the data for the surface dilational rheology vs. the applied frequency (Figure 6a,b). The dilational elasticities in the buffered solutions are systematically lower than in the ‘native’ pH system (Figure 6a). An interesting peculiarity is that minimal values are attained at pH = 5.04. This effect might be associated with structural peculiarities of the obtained LPS/tectomer complexes, related to the aging of the LPS stock solution, as well. The concentration of the stock solution is quite high (C(LPS) = 5.0 µg/L) and onsets of (pre)micellar entities are possible during the two-month initial incubation period, because of the sluggish nature of bulk LPS self-assembly [19,20]. These LPS species might be transferred in the T4+LPS aqueous samples, and could interact with the tectomers, and/or disintegrate and attach at the surface of the oligoglycine nano-platforms. Some of the LPS entities might be included in bulk “sandwich-like” motifs with the tectomers; the respective composites would not be surface active. The balance of these phenomena might also be related to the outcome that the surface dilational elasticity is higher at the lowest pH (3.80) and at highest pH (6.30) values, as compared to pH = 5.04 (Figure 6a).

The surface dilational viscosities are typically quite low and do not have a considerable pH-sensitivity, the values being almost the same as in the ’native’ pH case (Figure 6b). Anyway, higher values are attained only for pH = 5.04. Note that this is synchronous with the minimal values in the surface dilational elasticities for the same pH = 5.04. The reason for this behavior is still not fully clarified, and has to be further investigated. One might presume that either one-sided amphiphilic T4+LPS complexes which can build well-packed adsorption layer are predominantly formed, and/or two-sided amphiphilic T4+LPS complexes appear, which change the structure of the adsorption layer at the air/solution interface.

Overall, the adsorption-layer characteristics provide ample evidence for possible maximum number density in the onset of amphiphilic bulk complexes at pH = 5.04. At higher pH (6.30), the adsorption layer properties are almost as in the case of non-buffered solutions, most probably associated with a well-packed adsorption layer and a lower number of amphiphilic T4+LPS complexes in the subsurface region of the buffered solution. This notion is further supported by the results of surface dilational viscosities (Figure 6b). At lower pH (5.04), the viscosity values are higher, and they decrease at higher pH (6.30), reaching insignificant values similar to the ‘native’ pH case (5.70).

### 3.3. Effect of pH on the Drainage Peculiarities of Microscopic Foam Films

**Figure 7:**
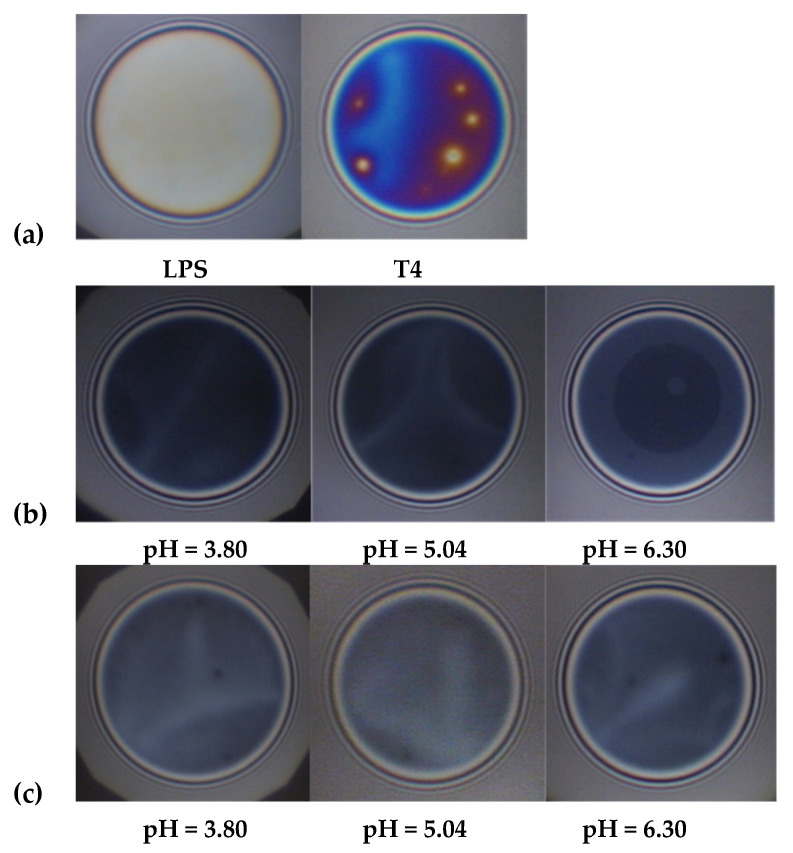
Characteristic film snapshots. (**a**) For ‘native’ pH solutions of LPS and T4; (**b**) for aqueous solutions of LPS at various pH values; (**c**) for T4+LPS mixtures at various pH values. The concentrations are C(T4) = 2 × 10^−4^ mol/L and C(LPS) = 0.5 µg/L. The initial incubation time of T4 solutions is 24 h; the incubation time of LPS stock solutions is 2 months. The temperature is t = 20 °C. Film radii are 100 µm.

**Figure 8:**
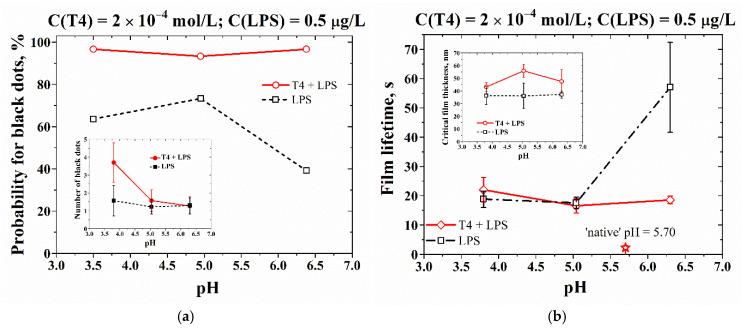
(**a**) Probability for the onset of black patterns in microscopic foam films, obtained from buffered aqueous solutions of LPS and of T4+LPS mixtures various pH values. C(T4) = 2 × 10^−4^ mol/L and C(LPS) = 0.5 µg/L. The initial incubation time of the T4 solutions is 24 h; the incubation time of LPS stock solutions is 2 months. The temperature is t = 20 °C. Film radius is 100 µm. The inset shows the results about the number of black patterns in the films against the pH values; (**b**) Lifetimes of microscopic foam films. The inset shows the respective results about the critical film thicknesses against the pH values.

Exemplary snapshots, illustrating the characteristic qualitative properties of microscopic foam films obtained from the LPS solutions, and from mixed T4+LPS systems, are presented in Figure 7a–c. The foam films from LPS aqueous solutions drain quickly with detectable darker spots (Figure 7b) and rupture in about ~20 s, except for pH = 6.30, when the film lifetime is about a minute (see also Figure 8b). This is in contrast to the LPS solutions of the same concentration C(LPS) = 0.5 µg/L at ‘native’ pH = 5.70 (Figure 7a). The foam films in the latter case are thick, have a uniform equilibrium thickness of ~102 nm, and do not rupture.

In the cases of buffered T4+LPS solutions (C(T4) = 2 × 10^−4^ mol/L, C(LPS) = 0.5 µg/L), the films drain rapidly within ~20 s; the respective snapshots evidence rather inhomogeneous film thicknesses, with the onset of dimples and black dots (Figure 7c). These outcomes are in contrast with the ‘native-pH’ case, where the films have well-outlined characteristic white patterns, with black dots in the middle of the white formations, and the obtained foam films are unstable, draining very quickly in less than 3 s (Figure 7a). As already commented in [29,30], the white patterns are due to the presence of (larger) tectomers in the film bulk, oriented parallel to the film interfaces, and they actually present thicker portions in the draining films. The black dots in the middle are related to secondary thinner film portions on top of the tectomeric aggregates. These patterns do not stabilize the foam films and they rupture in 1–3 s.

The results related to the probability of black pattern formation against the pH values of the buffered solutions are presented in Figure 8a. As is to be seen, in the case of mixed T4+LPS solutions, there is a considerable number of black patterns (spots and dots) at low pH which decrease at higher pH. This, however, does not significantly influence the probability of onset of these black patterns, which has no pH-dependency. There is no sensible impact on both the film lifetimes and the critical film thicknesses (Figure 8b).

Therefore, the foam films from aqueous solutions containing mixtures of T4 and trace quantities of LPS display most of the features that are well-known for films containing low molecular mass surfactants in the premicellar concentration range, namely rupturing films, short film lifetimes, and the onset of various film thickness irregularities [49,50]. However, the specific white patterns known from ‘native’ pH systems do not appear, while the characteristic black patterns are systematically observed (Figure 7). On the other hand, the latter do not substantially stabilize the foam films, and they drain rather quickly within 20–25 s (Figure 8b).

## 4. Discussion

**Scheme 2:**
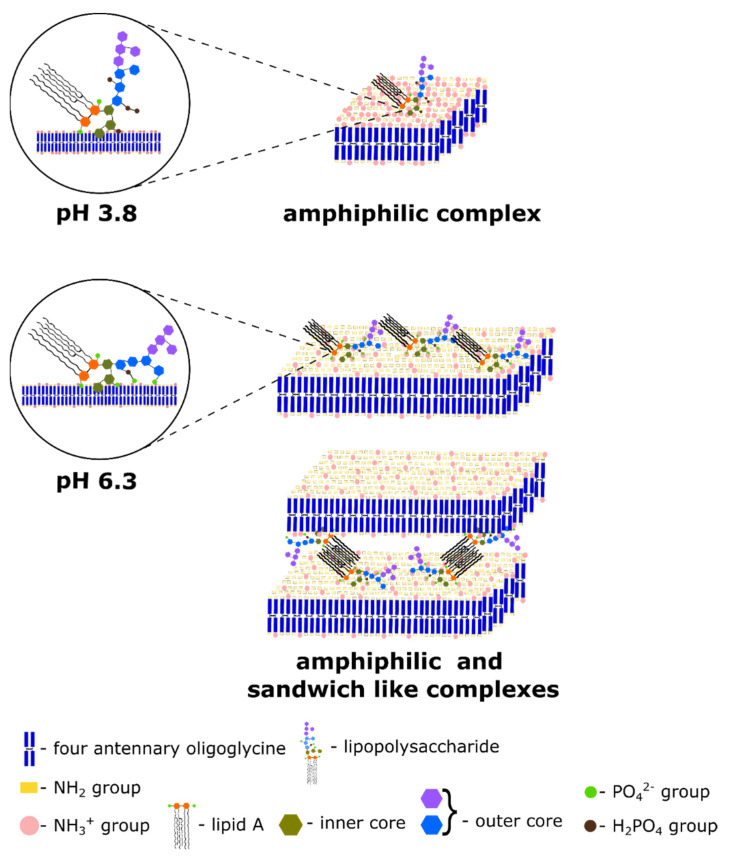
Schematic presentation of T4+LPS complexes at pH = 3.80 and pH = 6.30 in buffered aqueous solutions of T4+LPS mixtures. C(T4) = 2 × 10^−4^ mol/L and C(LPS) = 0.5 µg/L. The initial incubation time of T4 solutions is 24 h; the incubation time of LPS stock solutions is 2 months. The temperature is t = 20 °C.

The obtained results demonstrate the close interrelations between the bulk solution properties, the adsorption layer characteristics at the air/solution interface, and the drainage peculiarities of microscopic foam films for the investigated buffered T4+LPS aqueous systems at different pH values. The data from the DLS measurements give grounds for the hypothesis that, at constant T4 and LPS content, the pH-regulation affects predominantly the conditions for the onset of the bulk T4+LPS complexes. There are two major phenomena controlling this outcome:

(i) Upon increase of pH, and due to the quenching of the positive charge at the terminal amine groups in the oligoglycine antennae, conditions for the formation of larger bulk tectomers are created. Insofar as the quantity of LPS is very low, the detected size distributions of the T4+LPS complexes are defined by those of the T4 assembles within the whole range of pH-variations, as is to be evidenced by the thorough synchronization with the size distributions in the T4-only solutions (Figure 2).

(ii) The LPS and the oligoglycine entities are oppositely charged, and pH-regulation stimulates, in a different manner, the electrostatic interactions between the positively charged oligoglycine and the LPS entities, and affects the charge densities and the size distributions of the LPS molecules/premicelles, and of the T4 self-assemblies (tectomers). At lower pH, the T4 tectomers are smaller, and the effective electrical charges of the LPS species are diminished as compared to the ‘native’ pH conditions. So, the preference is for the formation of smaller bulk T4+LPS complexes, as particularly evidenced, for example, in Figure 2b,d. At higher pH (Figure 2e–h), the tectomers are larger, the charge of the LPS species is higher, and thus the T4 aggregates capture the LPS quantities more effectively in the aqueous bulk. So, the DLS results for the mixed systems virtually coincide with the T4-only size distributions. In addition, the specific interplay of the structure-interaction characteristics during the pH-regulation influences both the adsorption-layer properties, and the microscopic-foam-film behavior of the mixed buffered solutions of T4 and LPS. As already mentioned, it is assumed that the amphiphilic T4+LPS complexes are generated in the bulk of the solutions. The results in Figure 5a show that the trace quantities of LPS could not cause a detectable reduction of the surface tension values at the air/solution interface, as compared to air/water case [51]. While interacting with T4 tectomers, however, they may be responsible for the partial hydrophobization of the otherwise hydrophilic T4 assemblies. The formed amphiphilic T4+LPS complexes tend to adsorb at the air/solution interface, triggering the surface tension decrease. Because of the larger spatial areas taken by the tectomers with the attached LPS, the drop in the surface tension is significant in comparison with that of the LPS alone. The dynamic and equilibrium surface tension data for the mixed buffered systems are systematically higher than in the ‘native’ pH conditions. Besides, the equilibrium surface tension changes slightly by the pH-regulation, with a shallow minimum at ‘critical pH’ = 5.04 (Figure 5b). In order to explain this fact, an assumption is made for the possible formation of “sandwich-like” structures, as well (see also Scheme 2). The latter are constructed from disc-like T4 tectomers between which LPS molecules/assemblies could be trapped due to hydrophobic interactions, or LPS assembles interact with one or two tectomer flakes. The resulting T4+LPS complexes have external hydrophilic portions at both sides of the “sandwich” and would not possess surface activity. However, these entities keep the amphiphilic component (LPS) inactive, and remain in the aqueous bulk of the solutions, so that the surface tension would not be further decreased. At larger tectomer sizes and higher LPS charges (at pH = 6.30, Figure 5b), the equilibrium surface tension is even somewhat increased. So far, this assumption remains only a hypothesis, and requires further experimental confirmation.

The data about the surface rheology generally support this idea (Figure 6a,b). Thus, the surface dilational elasticity signals values similar to those in the range of premicellar concentrations of low-molecular-mass amphiphilic substances [49,50]. Interestingly, the surface dilational elasticity for T4+LPS solutions increases upon the increase of pH, approaching the values at ‘native’ pH (Figure 6a). This is in line with the notion about the increased capture of LPS in the solution bulk. In all cases, no substantial dependence on the frequency of the oscillating bubble is registered. As for the imaginary part of the surface elasticity modulus, namely the surface dilational viscosity, although it is quite low (less than 1 mN/m) for all investigated pHs, the values exhibit a maximum at the ‘critical pH’ = 5.04 (Figure 6b). The specific performance of the surface rheological characteristics might also be interpreted as the existence of an optimal pH-range ((5.04–6.30), for particular concentrations of T4 and LPS) within which the total entrapment of the LPS is achieved, both by the bulk (hydrophilic), and by the interfacial (amphiphilic) T4+LPS complexes.

The qualitative snapshots of the microscopic foam films (Figure 7b,c), and the formation of black patterns (Figure 8a) observed in the foam films of mixed T4+LPS solutions in particular, present additional evidence on the onset of amphiphilic T4+LPS complexes, because similar patterns are observed in surfactant systems containing premicellar structures [49,50]. However, due to the very low endotoxin quantity, the number of possible surface-active structures is not enough for the stabilization of the foam films, and they rupture rapidly within about 20 s. The foam film lifetimes drop substantially for the mixed T4+LPS systems, as compared to the LPS-only solutions. The minimum (~3 s) is registered for the ‘native’ pH case (Figure 8b). The destabilization of the films in the presence of the amphiphilic T4/LPS complexes might be generally regarded as an unusual result. However, similar film instability has been previously detected in two-component systems of the type polyelectrolyte/surfactant, with oppositely charged components [47,48]. The phenomenon was assumed to originate from the “rigid” mechanical properties of the complexes, which transforms the interfacial layers into harder and brittle coverage, and the films become very unstable. Due to the high level of PGII organization, tectomers appear more similar to non-flexible polyelectrolytes regarding their mechanical status than to soft assemblies (e.g., of micellar type for common surfactants). Thus, T4 aggregates could be considered as “rigid” and, as a consequence, their amphiphilic complexes with the endotoxin would not be able to create stable, compact and tight coverage in the adsorption layers at the air/solution interface, even if the amount of LPS entities in the solution is enough for this purpose. Therefore, each local area of the film free of coverage might be a potential rupture point.

## 5. Conclusions

The current study explores important aspects of a promising new approach for the detection and the entrapment of lipopolysaccharides from *E. coli* EH100 (LPS). The capture agents are the tectomers formed by synthetic four-antennary oligoglycine (C-(CH_2_-NH-Gly_7_)_4_, T4). Based on previously performed investigations of bulk and adsorption-layer properties, and of foam film drainage behavior of aqueous solutions containing T4 and LPS [30], the optimal conditions for the entrapment interactions are further fine-tuned by the pH-regulation of the aqueous systems.

The attained results show that, in the bulk of the aqueous solutions, tectomer/LPS complexes are generated. The initial reason for their formation is the electrostatic interactions between negatively charged portions of the LPS and the positively charged tectomeric aggregates. Earlier studies have shown that LPS is captured in amphiphilic T4+LPS complexes [29,30]. The present study reveals that, in the course of the pH-regulation of the fluid systems, and at fixed T4 and LPS quantities, two types of complexes are created: amphiphilic species and “sandwich-like” hydrophilic entities. At lower pH values, the complexes are smaller, at higher pH—they are larger. There is, however, a balance between the two types of complexes, so that the equilibrium surface tension data are actually quite similar within the whole range of pH variation. Besides, there is an optimal range of pH within which the whole quantity of the LPS is entrapped by the T4 self-assemblies, namely pH = 5.04–6.30.

It should be noted that the idea of the purification of aqueous solutions from endotoxins due to electrostatic binding to the most conserved portion of LPS molecules (Lipid A) has been implemented earlier in model studies, implying the use of affinity sorbents equipped with common ligands as deoxycholic acid, dimethylamine, histidine, polymyxin B, poly-ε-lysine and etc. [52,53]. In addition, nanoparticles and micro-particles decorated with cationic ligands have also shown considerable potential for biopharmaceutical purifications [54]. However, the approach described in the present study is simple and very flexible. The self-assembling nature of the components allows its fine-tuning and precise design of the aqueous formulation. Generally, it is also easier to remove the remaining capture agent and to regenerate it. The methodology is cost-effective because the synthesis of the four-antennary oligoglycine molecule is a relatively easy and inexpensive procedure. One very important advantage is that the T4 tectomers are biocompatible and void of any acute and chronic toxicity.

Overall, the data obtained in the present investigation back up the notion that the synthetic four-antennary oligoglycine C-(CH_2_-NH-Gly_7_)_4_ may be used as an effective identification and capture agent for traces of endotoxins in aqueous systems.

## Figures and Tables

**Table 1:** pH dependency of the Polydispersity Index (PdI)—(pH 3.8 – 8.05).

pH	PdI (T4)	PdI (LPS)	PdI (T4+LPS)
3.80	0.447 ± 0.062	0.577 ± 0.157	0.446 ± 0.091
5.04	0.516 ± 0.075	0.472 ± 0.123	0.539 ± 0.093
6.30	0.427 ± 0.020	0.379 ± 0.084	0.429 ± 0.033
7.26	0.394 ± 0.063	0.475 ± 0.151	0.208 ± 0.122
8.05	0.373 ± 0.118	0.431 ± 0.098	0.371 ± 0.128

## Data Availability

Data are available upon request.
